# Medical Department Grosvenor Library

**Published:** 1900-06

**Authors:** 


### MEDICAL DEPARTMENT GROSVENOR LIBRARY.

A VERY gratifying growth in the medical department of the Grosvenor Library is shown by the supplement to the catalogue of books in this department, recently published by the library. About 2,000 volumes and over 500 pamphlets were added during the year 1899, making a total January 1, 1900, of 5,316 volumes and 900 pamphlets.

This growth has been made possible by the liberality of the many friends of the library. The largest single donations were received from the New York Academy of Medicine and the University of Buffalo, but valuable accessions were also received from the libraries of a number of the physicians of Buffalo. A continuance of this generous interest on the part of physicians will soon place within their reach a very efficient medical library, enabling them to do work here for which they have hitherto had no conveniences nearer than New York or Chicago.

Although this collection is comparatively small, a good start has been made, and as the collection increases it is hoped that it may attract larger donations and bequests, and eventually become one of the important medical libraries of the country. The Grosvenor Library has furnished pleasant quarters for this department of its work, and a thorough and complete catalogue of all materials placed in its hand and is endeavoring, in so far as its means will allow, to procure for its shelves the new books as they appear. Physicians and other students of medicine are invited to use this collection freely, and to take a personal interest in its growth and improvement. Mr. E. P. VanDuzee, the efficient librarian, informs us that any suggestions for the purchase of books needed for special study, or the better arrangement of the material already in its possession, will be gratefully received by him, and will be favorably considered whenever circumstances permit.

The trustees very wisely and generously fitted up special apartments for the medical branch of the library, where physicians may read, annotate, and search the books and pamphlets undisturbed by the other visitors, and where they will find every convenience for conducting their researches. Such a library is a credit to the city, and to the officials who conduct it. Physicians should make free use of the opportunities which it affords, and thus testify their appreciation of its advantages.

